# Comparison of amyloid PET measured in Centiloid units with neuropathological findings in Alzheimer’s disease

**DOI:** 10.1186/s13195-020-00587-5

**Published:** 2020-03-04

**Authors:** Sanka Amadoru, Vincent Doré, Catriona A. McLean, Fairlie Hinton, Claire E. Shepherd, Glenda M. Halliday, Cristian E. Leyton, Paul A. Yates, John R. Hodges, Colin L. Masters, Victor L. Villemagne, Christopher C. Rowe

**Affiliations:** 1 Department of Molecular Imaging and Therapy, Austin Health, 145 Studley Road, Heidelberg, Vic. 3084 Australia; 2 CSIRO Health and Biosecurity, Parkville, Victoria 3052 Australia; 3 Victorian Brain Bank, The Florey Institute of Neuroscience and Mental Health, Melbourne, Australia; 4 Sydney Brain Bank, Neuroscience Research Australia and Faculty of Medicine, University of NSW, Sydney, Australia; 5 The Brain and Mind Centre, Faculty of Medicine and Health, University of Sydney, Sydney, Australia

**Keywords:** Amyloid imaging, Alzheimer’s disease, Centiloids, Positron emission tomography, Neuropathology

## Abstract

**Background:**

The Centiloid scale was developed to standardise the results of beta-amyloid (Aβ) PET. We aimed to determine the Centiloid unit (CL) thresholds for CERAD sparse and moderate-density neuritic plaques, Alzheimer’s disease neuropathologic change (ADNC) score of intermediate or high probability of Alzheimer’s Disease (AD), final clinicopathological diagnosis of AD, and expert visual read of a positive Aβ PET scan.

**Methods:**

Aβ PET results in CL for 49 subjects were compared with post-mortem findings, visual read, and final clinicopathological diagnosis. The Youden Index was used to determine the optimal CL thresholds from receiver operator characteristic (ROC) curves.

**Results:**

A threshold of 20.1 CL (21.3 CL when corrected for time to death, AUC 0.97) yielded highest accuracy in detecting moderate or frequent plaque density while < 10 CL was optimal for excluding neuritic plaque. The threshold for ADNC intermediate or high likelihood AD was 49.4 CL (AUC 0.98). Those cases with a final clinicopathological diagnosis of AD yielded a median CL result of 87.7 (IQR ± 42.2) with 94% > 45 CL. Positive visual read agreed highly with results > 26 CL.

**Conclusions:**

Centiloid values < 10 accurately reflected the absence of any neuritic plaque and > 20 CL indicated the presence of at least moderate plaque density, but approximately 50 CL or more best confirmed both neuropathological and clinicopathological diagnosis of Alzheimer’s disease.

**Supplementary information:**

**Supplementary information** accompanies this paper at 10.1186/s13195-020-00587-5.

## Background

Current standard-of-truth (SoT) diagnosis of Alzheimer’s disease (AD) largely depends on neuropathological demonstration of brain amyloid-beta (Aβ) plaques and tau tangles [1]. Recently, research criteria for detection of AD have emphasised the importance of amyloid and tau positron emission tomography (PET) imaging biomarkers [2, 3].

^11^C-Pittsburgh Compound B (^11^C-PiB), ^18^F-florbetaben (FBB), ^18^F-florbetapir, ^18^F-flutemetamol and ^18^F-NAV4694 (NAV) are PET tracers that demonstrate binding to brain Aβ in AD from the preclinical AD stage onwards, with good sensitivity and specificity as biomarkers of antemortem AD pathology and predictors of progression to AD dementia [4–10]. Amyloid PET scans are used for inclusion and monitoring in AD-modifying clinical therapy trials and to aid clinical diagnosis and prognostication [11, 12].

Variability in tracers, PET scanners, procedural factors, and analysis methods across imaging centres have driven attempts for quantitative standardisation of amyloid PET results. Klunk and colleagues [13] derived a scale of “Centiloid” units (CL) for standardised reporting of amyloid imaging. CL values range beyond the “anchor-point” of 0, representing young healthy controls, and 100, representing the amyloid burden present in average mild to moderate severity dementia due to AD. This important work allows for amyloid PET scans across different sites to yield comparable data.

Comparison of neuropathological data with positive or negative amyloid PET scans based on expert visual read has been performed for ^11^C-PiB, ^18^F-florbetapir, ^18^F-florbetaben and ^18^F-flutemetamol [7, 9, 14–18]. In vivo biomarkers such as cerebrospinal fluid Aβ, tau PET, and volumetric MRI have been compared with amyloid PET in CL [19, 20]. Three recent studies have examined the performance of amyloid PET CL thresholds compared with SoT neuropathology. These studies reported thresholds for detection of moderate or frequent neuritic plaque ranging from 12 to 24 CL but did not correct for time elapsed between amyloid scan and death and had relatively few cases with CL values close to the threshold values [21–23]. Only one compared to Alzheimer’s disease neuropathologic change (ADNC) rating [1] or to expert visual read report of a positive scan [21].

We aim to further define the accuracy of CL values when compared with SoT post-mortem neuropathological data on neuritic amyloid plaque density, ADNC rating, final clinicopathological diagnosis of AD, and visual reading threshold for a positive amyloid PET scan.

## Methods

### Compliance with ethical standards

Ethics approval was obtained from the Austin Health Human Research Ethics Committee (reference LNR/17/405).

### Subject identification and demographic data collection

Fifty-one subjects in total with various ante-mortem and post-mortem diagnoses were retrospectively identified by cross-referencing the databases of the Austin Health Molecular Imaging Dementia Research group, the Sydney Brain Bank, and the Victorian Brain Bank. These subjects, with prior informed consent, had undergone both an amyloid PET scan at Austin Health and post-mortem neuropathologic brain evaluation in Melbourne or Sydney between all years recorded in the database (2004 to 2017). Exclusion criteria for these prior studies were history of stroke, significant medical illness, recent cancer, and substance use disorder. Two of the 51 cases were excluded due to a diagnosis of familial AD, because different neuropathological processes in this condition, such as a significantly greater density of Aβ plaques in the cerebellum than in sporadic AD [24, 25], may have confounded amyloid PET quantification and interpretation. Data has been published on a proportion of the cases [26, 27].

### Amyloid PET imaging and Centiloid determination

Aβ imaging was performed with either ^11^C-PiB or ^18^F-florbetaben (FBB). The methodology for PET imaging with these tracers has been previously described [28, 29]. A 20-min acquisition was commenced 50 min post-injection of ^11^C-PiB or 90 min post-injection of FBB. A transmission scan was performed for attenuation correction. PET images were reconstructed using a 3D row-action maximum likelihood algorithm (RAMLA). The standard Centiloid cortical and whole cerebellar volumes of interest template were applied to the summed and spatially normalised PET images in order to obtain standardised uptake value ratios (SUVR). For this study, we used the CapAIBL software package, which when compared to standard approach has the benefit of not requiring a corresponding MRI to quantify the PET scan [30, 31]. This package has been validated against the standard Centiloid method that uses the public domain software program SPM8 to spatially normalise each subject’s MRI and then apply those parameters to spatially normalise the amyloid PET scan [30]. The SUVR were transformed into Centiloid units by linear transformation using the PET tracer-specific equations published for conversion of Centiloid method SUVR to Centiloid units with a minor correction applied for the CapAIBL registration [13, 28, 29, 32].

### Neuropathologic evaluation

Neuropathological evaluation was performed at the Victorian Brain Bank (Melbourne, Australia) and Sydney Brain Bank (Neuroscience Research Australia, Sydney, Australia) to determine a global C score from inferior temporal regions of fixed brain hemispheres based on the Consortium for Establish a Registry for Alzheimer’s Disease (CERAD) neuropathologic assessment guidelines [33]. Frequency of neuritic plaques per ×100 microscopic field were categorised as none, sparse, moderate or frequent with corresponding C scores of 0, 1, 2 or 3 respectively, as described in published guidelines [34]. ADNC classification was also obtained as defined by the NIA-AA 2012 criteria [1]. The ADNC rating uses the Thal amyloid plaque distribution, the Braak neurofibrillary tangle stage and the CERAD neuritic plaque score, to classify AD neuropathologic change as not, low, intermediate or high.

### Visual read

One amyloid PET expert reader (author CR), blinded to CL values and neuropathological data, visually interpreted all scans using MedView v12 software, viewing images in greyscale and rainbow colour scale. The method used to visually read amyloid PET has been previously described [35]. Scans were classified positive when cortical activity was equal to or greater than white matter activity in one or more lobes.

### Clinicopathological diagnosis

The clinicopathological diagnosis for each case factored in both neuropathological assessment and antemortem clinical diagnosis. Neuropathological diagnosis was made in accordance with the published guidelines [34] and included morphological examination with immunohistochemistry analyses for Aβ, tau, TDP43 and alpha-synuclein in several brain regions. There were 17 AD and 32 non-AD cases. Non-AD cases included diagnoses of frontotemporal dementia (*n* = 12), normal controls (*n* = 3), dementia with Lewy bodies (*n* = 3), Parkinson’s disease dementia with concurrent diffuse Lewy bodies (*n* = 3), hippocampal sclerosis (*n* = 2), Creutzfeldt-Jakob disease (*n* = 2), progressive supranuclear palsy (*n* = 2), motor neuron disease (*n* = 1), hippocampal ischaemia (*n* = 1), corticobasal degeneration (*n* = 1), multisystem atrophy (*n* = 1) and a case of mixed AD and dementia with Lewy bodies (*n* = 1). This last case was included in all analyses, except for the “Centiloid results in clinicopathological AD diagnosis” analysis.

### Statistical analyses

Three aspects of CL performance were investigated. Firstly, CL values were compared with dichotomized neuropathological C score categories using two different approaches: “high vs low” plaque density (“high” = moderate and frequent, and “low” = none and sparse) and “any vs none” (“any” = sparse, moderate and frequent, and “none” = none). A Youden index [36] was used to determine the optimal CL thresholds from receiver operator characteristic curves. CL values were also compared with binary ADNC classification of “unlikely AD” (ADNC scores of not or low) vs “likely AD” (ADNC scores of intermediate or high). Secondly, values were compared with visual read (positive or negative). Thirdly, CL values were compared with cases of AD as determined by clinicopathological diagnosis using descriptive statistics. To assess for the contribution of interval from PET scan to time to death, analyses were repeated using adjusted CL values, after applying a sigmoidal adjustment derived from our previous work [37]. We derived that CL increases very slowly below 20 CL, then accelerates to a maximum of 5 CL increase per year for the almost linear mid-section (40 to 110 CL) of the sigmoid curve that best describes amyloid accumulation over time, before slowing again at higher CL values. Consequently, each individual CL value was adjusted to that expected at the time of death based on the average rate of increase for the CL level at the time of the scan and the duration between the scan and post-mortem examination.

## Results

### Case characteristics

Of the 49 included subjects, 33 underwent ^11^C PiB PET and 16 underwent FBB PET. Thirty-eight (78%) cases were male. The mean age at death was 76 years, and the median interval between date of last amyloid PET scan and death was 2.75 years (IQR ±3.05, range 0.03 to 5.64). Twenty-five subjects had a C score of 0 (no plaques), five had a C score of 1 (sparse plaques), five had a C score of 2 (moderate plaques) and 14 had a score of 3 (frequent plaques). Twenty had ADNC classification of intermediate or high neuropathologic change.

### Centiloid results and neuropathological C score categories

**Fig. 1:**
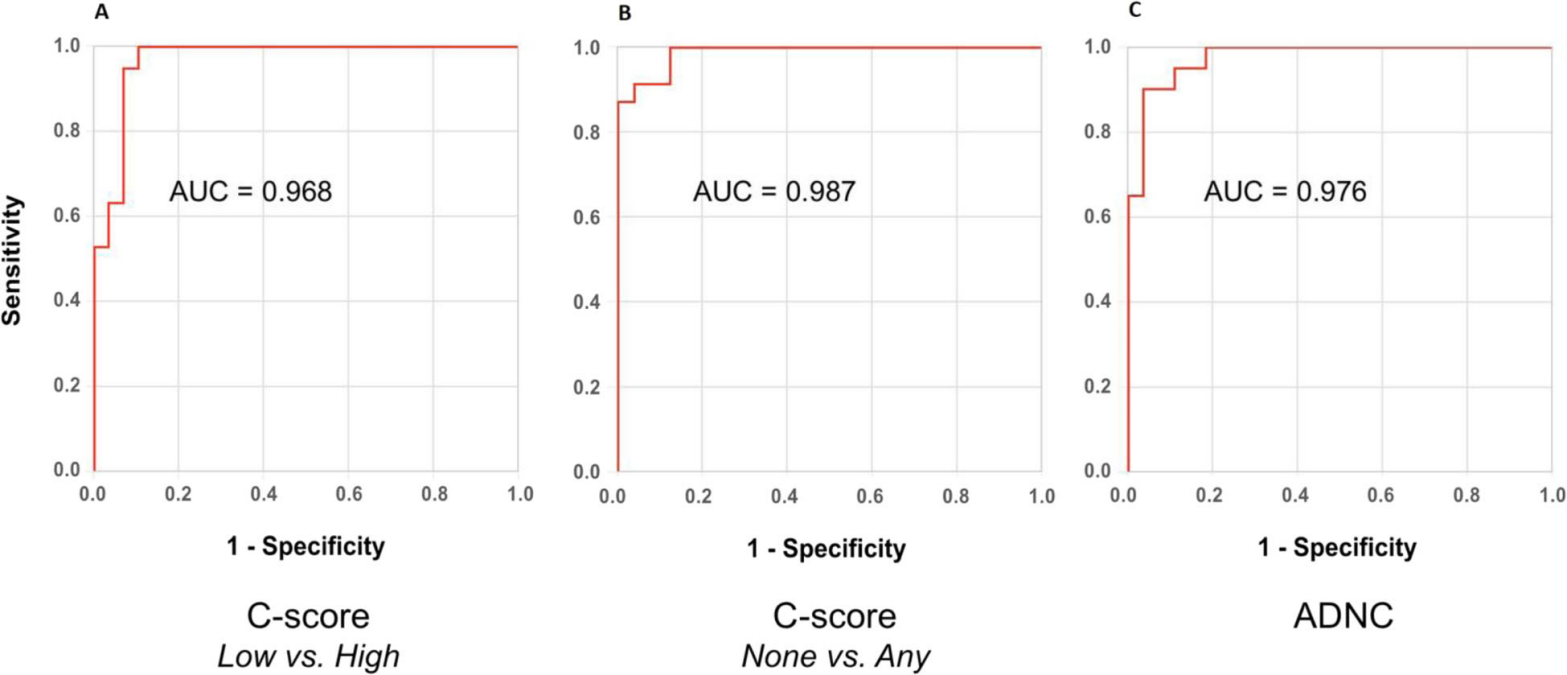
ROC curves: corrected Centiloids and neuropathologic results. Receiver Operator Characteristic curve for corrected Centiloid (CL) units thresholds in determining **a**) “low” vs “high” C score neuritic amyloid plaque burden (optimal threshold 21.3 CL, Youden J= 0.893) ; **b**) “none” vs “any” C score neuritic amyloid plaque burden (optimal threshold 9.6 CL, Youden J= 0.875); and **c**) “unlikely Alzheimer’s disease” (not/low scores) vs “likely Alzheimer’s Disease” (intermediate/high scores) using Alzheimer’s Disease Neuropathologic Change evaluation (optimal threshold 49.4 CL, Youden J= 0.863)

**Fig. 2:**
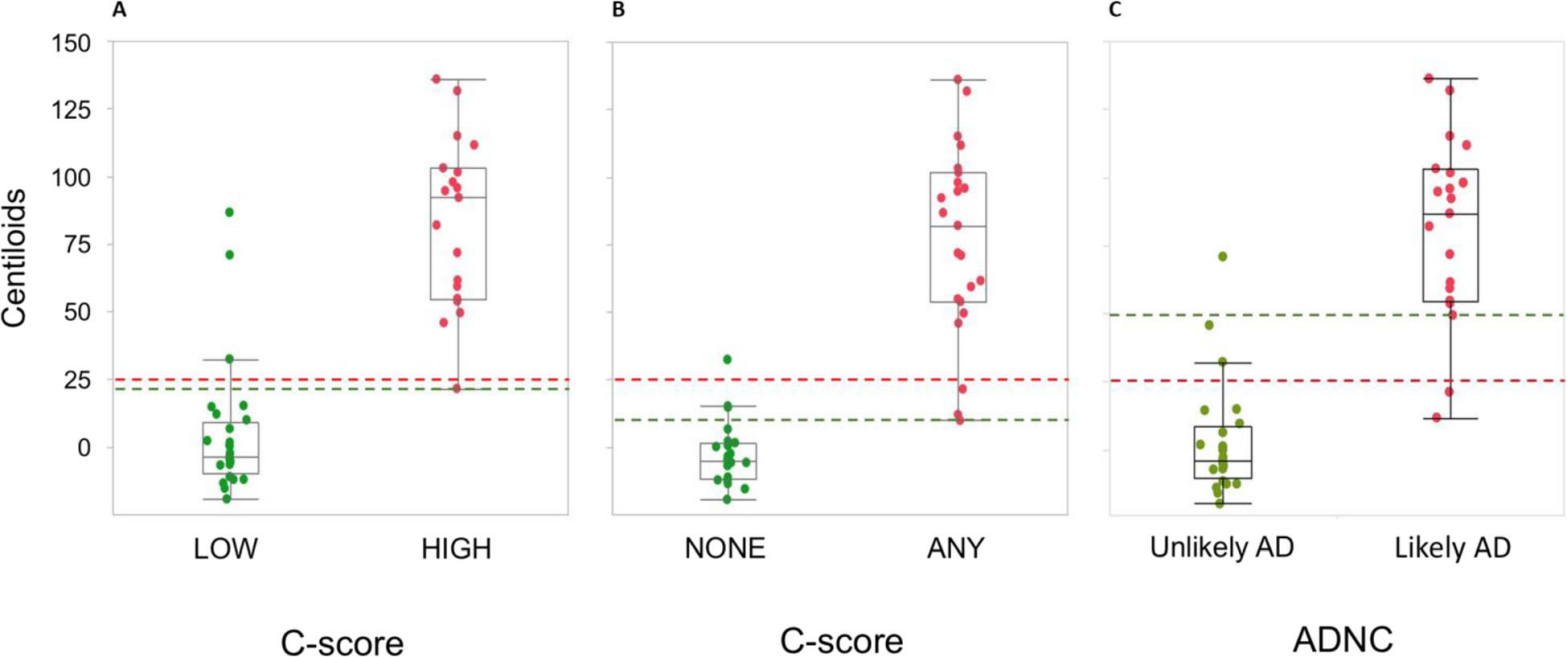
Scatterplots: corrected Centiloids and neuropathologic results. Scatterplots for corrected Centiloids against **a**) “low” vs “high” C score neuritic amyloid plaque burden, **b**) “none vs “any” C score neuritic plaque burden, and **c**) “unlikely AD” and “likely AD” using Alzheimer’s Disease Neuropathologic Change evaluation scores. The red dashed line denotes the 25 Centiloid (CL) mark, and the green dashed lines denote the thresholds of A) 21.3 CL; B) 9.6 CL; and C) 49.4 CL

There were 19 (39%) patients with “high” and 30 (61%) with “low” C scores. The receiver operator characteristic curve demonstrated an optimal threshold of 20.1 CL for detection of a high level of amyloid plaque (i.e. moderate or frequent neuritic plaques). After applying the sigmoidal adjustment for interval from scan to post-mortem, the putative optimal threshold was 21.3 CL (Fig. 1a). Data points and threshold are shown in Fig. 2a.

When neuritic plaque scores were grouped as “any” (i.e. sparse or more) vs “none”, there were 24 (49%) cases with “any” and 25 (51%) with no neuritic plaques. The optimal CL threshold found for detecting the presence of any amyloid plaques (i.e. sparse or more) was 9.5 CL. After applying the sigmoidal adjustment for interval from scan to post-mortem, the putative optimal threshold was 9.6 CL (Fig. 1b). Data points and threshold are shown in Fig. 2b.

### Centiloid results and ADNC diagnosis

There were 20 (41%) patients with intermediate to high probability of AD. The optimal threshold found for detection of intermediate to high ADNC was 46.9 CL. After applying the sigmoidal adjustment for interval from scan to post-mortem, the putative optimal threshold was 49.4 CL (Fig. 1c). Data points and threshold are shown in Fig. 2c.

### Centiloid results and amyloid PET visual read

**Fig. 3:**
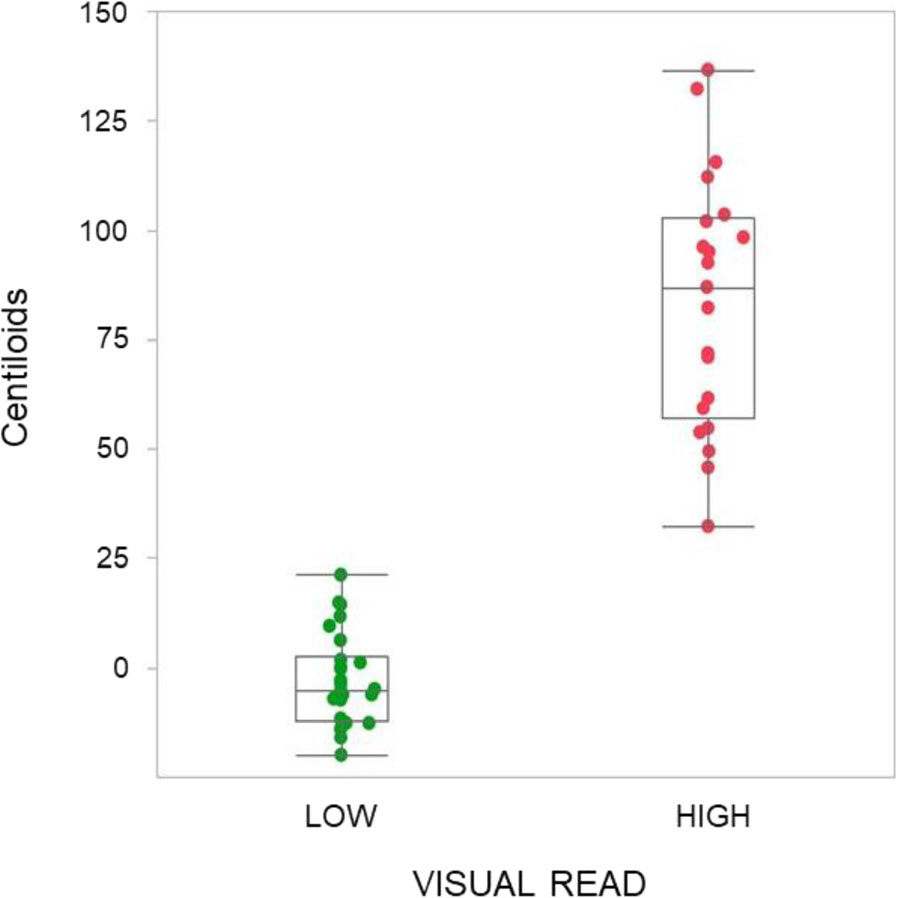
Centiloid results and amyloid PET visual read ratings. Scatterplot for Centiloid unit threshold testing against binary expert visual read categories. A 26 CL cut-off yielded a 100% match to expert visual read of “high” or “low”

Correlation of Centiloid values with amyloid PET expert visual read (positive or negative) yielded an AUC of 1.0 and optimal CL threshold of 26. Using this threshold, there was 100% agreement between Centiloid (elevated/not elevated) and visual read (positive/negative) (Fig. 3). The effect of the visual read threshold which equates to 26 CL for detection of moderate or frequent plaque compared to the quantification best threshold of 20.1 CL is a minor reduction from 1.0 to 0.95 in sensitivity and from 1.0 to 0.90 in specificity.

### Centiloid results in clinicopathological AD diagnosis

CL values were reviewed for the 17 cases that had a final clinicopathological diagnosis of AD. The median CL result was 87.7 (IQR ± 42.2), and 16 of the 17 cases (> 90%) had a value of > 45 CL. In contrast, the median CL result in the 11 cases with a clinicopathological diagnosis of FTD was − 4.9 CL.

## Discussion

Improving accuracy of the detection of brain amyloid plaques is important for clinical trial enrichment in the quest to develop disease-modifying or curative treatment for the growing burden of Alzheimer’s disease. Contemporary clinical application can also better assist diagnosis, prognostication and planning for patients with cognitive disorders.

We have demonstrated that a threshold of 20.1 CL was optimal for the detection of “high” levels of neuritic plaque density, as determined by C score moderate or frequent classification. In other words, values of 20.1 CL or lower accurately reflected the absence of moderate or frequent plaques, with a high AUC. This threshold was not significantly altered when corrected for time between scan and post-mortem. CL results below this threshold should provide reassurance that patients are unlikely to have Alzheimer’s disease. This is reasonably concordant with the findings of Navitsky and colleagues, who determined a threshold of 24.1 CL with florbetapir for CERAD amyloid plaque classification of moderate or frequent vs sparse or none, in 59 individuals [22]. This is also concordant with the Centiloid analysis by Dore and colleagues of a florbetaben phase III post-mortem study in 66 individuals, which yielded a threshold of 19 CL for the same categorisation [23]. These thresholds are higher than those identified by La Joie et al., who determined a threshold of 12.2 CL for separating none or sparse plaques from moderate or frequent plaques; but oddly they found the same threshold for separating any plaques from no plaques in 179 individuals scanned with ^11^C-PiB PET [21]. Those authors did, however, suggest 24 CL to be a more appropriate threshold for “identifying clinically meaningful Aβ burden” based on ADNC neuropathological criteria and Thal scores. These thresholds may be useful as cut-offs for clinical trial enrichment, or for guiding decision-making about commencing potential disease-modifying therapies when they become available.

For the detection of “any” amyloid plaque (sparse or more), the optimal threshold identified was 9.5 CL. Once again, this value only marginally increased when corrected for time between scan and post-mortem. This threshold is similar to two standard deviations of young controls as determined by Klunk and colleagues, equalling 8.68 CL [13] and 12.2 CL [21] for PiB and FBB respectively. This suggests there are no differences in amyloid tracer binding between young and old individuals when no amyloid plaques are present, indicating no substantial increase in non-specific binding of these tracers nor significant changes in tracer kinetics with normal ageing.

A threshold of 26 CL exactly matched expert visual read of positive vs negative scan. This is consistent with good concordance (97%) noted by Leuzy and colleagues [19] between visual read and PiB CL results. This visual threshold is slightly higher than the CL threshold for neuropathology indicating that some persons with significant AD pathology will have a visually negative scan and that scan quantification is of value when identifying persons with AD pathology for clinical trials.

Our expert visual reader results are comparable to those reported in several phase III trials of PET tracers that compared blinded expert visual reads of amyloid imaging with post-mortem data, where moderate to frequent neuritic plaques (a “high” classification in our study) were considered positive. Florbetaben visual reads were reported as having sensitivity of 97.9% and specificity of 88.9% [16]. Florbetapir visual reads yielded sensitivity of 92% and specificity of 100% within a 2-year window between imaging and autopsy [15]. Flutemetamol visual reads in one study correlated with both original and modified CERAD criteria, yielded respective sensitivities of 91.9% and 90.8%, and specificities of 87.0% and 90.0% [17]. Three expert readers in La Joie’s group [21] had 99% accuracy for scans above a threshold of 24 CL. When considering visual read, there are rare cases of clear focal amyloid uptake that may lead to a positive visual read but a low CL score but there were no such cases in our cohort.

In our cases with clinicopathological AD, a median CL value of 87.7 was found at post-mortem, but with significant variability as demonstrated by the IQR of ± 42.2 CL. Only one of these cases, scoring 20.1 CL (21.3 CL when corrected for time to death), returned a result under 45 CL, suggesting that a sensitivity cut-off for defining clinicopathological AD should be considerably higher than that for detecting “high” amyloid plaques alone. This agrees well with our finding of 49 CL as the optimal threshold for identifying cases that meet current neuropathological criteria for AD based on a comprehensive post-mortem brain examination, i.e. intermediate or high Alzheimer’s Disease Neuropathic Change (ADNC). Our exceptional case with only 21 CL had logopenic aphasia and 5.6 years between ^11^C-PiB scan and death at which time frequent plaques were found. The PiB scan was reviewed and showed mild patchy cortical binding. The long interval between scan and post-mortem is note-worthy and raises the possibility of more rapid than usual plaque accumulation so that the correction for time elapsed from scan did not provide an accurate estimation of the plaque burden at the time of death. Our CL findings in clinicopathological AD compare to estimates obtained without pathological confirmation of AD. For instance, Leuzy et al. [19] demonstrated median PiB PET results of 47.5 CL for mild cognitive impairment and 84.1 CL for AD in their cohorts. We reviewed the 230 patients with a diagnosis of probable AD made by a clinical panel blinded to biomarker findings including amyloid PET in the Australian Imaging Biomarkers and Lifestyle study of ageing. The mean CL in these patients was 95 ± 30. Larger numbers in future studies would help confirm if a clinicopathological diagnosis of AD is indeed rare when under 45 CL.

The sex distribution in this study was predominantly male at 78%. No explanation for this is evident in our study, but a predominance of males was also reported in two of the three published CL neuropathological correlation studies [21, 22].

Our study adds further confirmation of these thresholds in a field where there are relatively low numbers of post-mortem results for correlation, especially for intermediate CL values. Additionally, we have adjusted for amyloid accumulation during the time between scan and post-mortem. Finally, only La Joie and colleagues [21] have also included a visual read comparison in a cohort with neuropathological assessment.

### Limitations

A limitation of the study is the data distribution, in that only two subjects had results between 15 and 35 CL, consequently restricting the ability to tightly define thresholds. Specifically, these subjects had results of 20.1 and 30.9 CL.

Another limitation is the time elapsed between scan and death. This averaged approximately 3 years. We have accounted for this using a correction based on the published curve for amyloid accumulation. However, this correction had minimal effect on the study findings. Familial AD cases were excluded from this study due to potential presence of neuritic amyloid plaques in the cerebellum [24], and the lower affinity of PiB to “cotton wool” plaques found in some presenilin mutations [38]. These cerebellar plaques could interfere with the scaling to SUVR and return misleading low CL results not applicable to typical sporadic AD [25]. Separate characterisation of CL performance in familial AD is warranted.

## Conclusions

In our cohort, values < 9.5 CL accurately reflected the absence of any neuritic plaques, and > 20.1 CL indicated the presence of at least moderate plaque density. These neuropathology-based Centiloid thresholds may be used to exclude a diagnosis of AD and to define groups for early intervention and other disease specific trials. Approximately 50 CL or more best confirmed both neuropathological and clinicopathological diagnosis of Alzheimer’s disease.

## Supplementary information

**Additional file 1.** Supplementary data tables.

## Abbreviations

^11^C-PiB: ^11^C-Pittsburgh Compound B
AD: Alzheimer’s disease
ADNC: Alzheimer’s disease neuropathologic change
AUC: Area under the curve
Aβ: Beta-amyloid
CERAD: Consortium for Establish a Registry for Alzheimer’s Disease
CL: Centiloid
FBB: ^18^F-florbetaben
IQR: Interquartile range
MRI: Magnetic resonance imaging
NAV: ^18^F-NAV4694 (NAV)
PET: Positron emission tomography
RAMLA: Row-action maximum likelihood algorithm
ROC: Receiver operator characteristic
SoT: Standard of truth
SUVR: Standardised uptake value ratio
TDP43: TAR DNA-binding protein 43
